# Novel Bioactive and Therapeutic Dental Polymeric Materials to Inhibit Periodontal Pathogens and Biofilms

**DOI:** 10.3390/ijms20020278

**Published:** 2019-01-11

**Authors:** Minghan Chi, Manlin Qi, Lan A, Ping Wang, Michael D. Weir, Mary Anne Melo, Xiaolin Sun, Biao Dong, Chunyan Li, Junling Wu, Lin Wang, Hockin H. K. Xu

**Affiliations:** 1 Department of Oral Implantology, School of Dentistry, Jilin University, Changchun 130021, China; minghan93@hotmail.com (M.C.); qml1992@126.com (M.Q.); hi_alan2001@163.com (L.A); sxl2673366@126.com (X.S.); 2 Jilin Provincial Key Laboratory of Sciences and Technology for Stomatology Nanoengineering, Changchun 130021, China; 3 Department of Advanced Oral Sciences and Therapeutics, University of Maryland School of Dentistry, Baltimore, MD 21201, USA; dentistping@hotmail.com (P.W.); mweir@umaryland.edu (M.D.W.); mmelo@umaryland.edu (M.A.M.); hxu@umaryland.edu (H.H.K.X.); 4 State Key Laboratory on Integrated Optoelectronics, College of Electronic Science and Engineering, Jilin University, Changchun 130012, China; dongb@jlu.edu.cn; 5 Shandong Provincial Key Laboratory of Oral Tissue Regeneration, Department of Prosthodontics, School of Stomatology, Shandong University, Jinan 250012, China; 6 Center for Stem Cell Biology and Regenerative Medicine, University of Maryland School of Medicine, Baltimore, MD 21201, USA; 7 University of Maryland Marlene and Stewart Greenebaum Cancer Center, University of Maryland School of Medicine, Baltimore, MD 21201, USA

**Keywords:** polymers, antibacterial, drug delivery, periodontitis, periodontal biofilms

## Abstract

Periodontitis is a common infectious disease characterized by loss of tooth-supporting structures, which eventually leads to tooth loss. The heavy burden of periodontal disease and its negative consequence on the patient’s quality of life indicate a strong need for developing effective therapies. According to the World Health Organization, 10–15% of the global population suffers from severe periodontitis. Advances in understanding the etiology, epidemiology and microbiology of periodontal pocket flora have called for antibacterial therapeutic strategies for periodontitis treatment. Currently, antimicrobial strategies combining with polymer science have attracted tremendous interest in the last decade. This review focuses on the state of the art of antibacterial polymer application against periodontal pathogens and biofilms. The first part focuses on the different polymeric materials serving as antibacterial agents, drug carriers and periodontal barrier membranes to inhibit periodontal pathogens. The second part reviews cutting-edge research on the synthesis and evaluation of a new generation of bioactive dental polymers for Class-V restorations with therapeutic effects. They possess antibacterial, acid-reduction, protein-repellent, and remineralization capabilities. In addition, the antibacterial photodynamic therapy with polymeric materials against periodontal pathogens and biofilms is also briefly described in the third part. These novel bioactive and therapeutic polymeric materials and treatment methods have great potential to inhibit periodontitis and protect tooth structures.

## 1. Introduction

Periodontitis is a dental plaque (bacteria)-induced, and host-mediated, breakdown of soft and hard tissues surrounding the teeth [1]. It is a persistent disease with no appreciable decrease in the prevalence of periodontitis in any of the world’s regions between 1990 and 2010 [2]. In 1999, nearly 35% of individuals ≥30 years of age showed signs of periodontitis in the United States [3]. Severe periodontitis was reported as the sixth most prevalent chronic condition in 2010, affecting 10.8% of the population 15–99 years of age, or 743 million people [2]. Furthermore, the prevalence of periodontal disease increases with age. More than 70% of adults aged 65 or older are diagnosed with some form of periodontal disease [4]. 

Microbial biofilms are the aggravative factor of periodontitis [5]. The conventional treatment for periodontitis involves mechanical processing (such as supragingival scaling, subgingival scaling, and root planing) advantageously accompanied by the adjuvant administration of antibiotics, which can be applied by systemic or local administration [6]. The periodontal pocket provides a natural reservoir, which is easily accessible for the insertion of a delivery device. Therefore, intra-pocket drug delivery systems are highly desirable due to the potentially lower incidence of undesirable side effects, improved efficacy and enhanced patient compliance. Recent progress in polymer sciences have produced bioactive polymeric materials, which can be modified to meet pharmacological and biological requirements [7]. First, polymers can possess strong antibacterial properties against periodontal pathogens and could be used as an alternative to low molecular weight antimicrobial agents in periodontitis treatment due to their low cost, versatility, and processability [8,9,10,11]. Second, polymeric materials can serve as intra-pocket drug delivery devices for the treatment of periodontitis in various formulations and forms. Third, polymeric materials can be applied in fabrication of biodegradable periodontal membranes for guided tissue regeneration (GTR) [12,13,14,15,16]. The periodontal membrane acts as a mechanical barrier which protects the clot, and allows periodontal ligament and bone tissue to selectively repopulate the root surface during healing [12]. The main risk that contributes to unsuccessful tissue regeneration by GTR membrane treatment is the action of periodontal pathogens. Therefore, multifunctional GTR membranes were recently developed to possess not only barrier and tissue regenerative properties, but also antibacterial effects against periodontal pathogens. Several meritorious reviews on polymeric materials have described their antibacterial effects against pathogenic microorganisms [17,18,19], drug delivery capabilities [20,21,22] and periodontal regeneration for periodontitis treatment [23,24,25], which is not repeated here.

Tooth-colored polymeric composites and bonding agents are the primary materials for restoring tooth cavities [26,27,28,29,30]. This is because advances in polymer chemistry and filler particle compositions have enhanced the composite restoration properties [31,32,33,34,35,36]. As the world population ages, there is an increasing trend of root caries in senior people. Root caries can be treated with Class-V restorations. However, they often have subgingival margins which are difficult to clean and can provide pockets for periodontal bacterial growth. This in turn leads to the worsening of periodontitis and the damage of the periodontal attachment. To make matters worse, the currently available dental polymer-based Class-V composites not only have no antibacterial effect, but actually accumulate more biofilms and plaque than other materials such as metals. The present article reviews new developments in polymeric materials for periodontitis treatment, periodontal tissue regeneration and Class-V restorations, focusing on bioactive and therapeutic effects against periodontal pathogens and biofilms.

## 2. Polymeric Materials as Drug Carrier for Combating Periodontal Biofilm

The origin of periodontitis is closely related with a dramatic shift from a symbiotic microbial community to a dysbiotic microbial community that is mainly composed of anaerobic genera [37]. As the treatment of periodontitis mainly focuses on the elimination of the periodontal pathogens or biofilms from the tooth surface, antibiotics are commonly used to treat the disease. Therefore, intra-pocket drug delivery systems are highly desirable because they can maintain effective high levels of antibiotics in the gingival crevicular fluid for a prolonged period of time [7]. Meanwhile, they have lower risks of undesirable side effects, superior efficacy and enhanced patient compliance [7]. Due to the relatively low price, higher stability, nontoxicity, biocompatibility, nonimmunogenicity and biodegradability, a variety of polymers are investigated as drug carriers in various formulations and forms such as fibers [38,39,40,41], strips [42], films [43,44], gels [45,46], micro-particles [47,48,49], nanoparticles [11,50,51,52], and vesicular system [53,54]. Table 1 shows various polymer-based local drug delivery systems delivering a variety of antibiotics, such as metronidazole, doxycycline, tetracycline and secnidazole, for combating periodontal pathogens. 

Gels as semisolid systems could easily apply to the site of action with fast drug release rate, adhere to wide area of mucosa in the dental pockets, and maintain the concentration of antibiotics due to their bio-adhesive characteristics [69]. Polymers such as chitosan, poly (dl-lactide-co-glycolide) (PLGA) and polyacrylic acid are used for the preparation of gels. Gad et al. formulated PLGA “in situ implants” containing secnidazole and doxycycline by a non-solvent-induced phase separation mechanism [56]. The antibacterial evaluation against *Porphyromonas gingivalis* (*P. gingivalis*) showed that implants with 25% PLGA showed a greater zone of inhibition than a commercial control Atridox^®^, which is a locally delivered antimicrobial product containing doxycycline hyclate [56].

In addition, films are widely used for intra-pocket delivery. Films could be fabricated either by solvent-casting or direct milling. The drugs are distributed throughout the polymer and released by diffusion and/or matrix dissolution or erosion [70]. Importantly, the shape of the film can be easily adjusted according to the dimensions of the intra-pocket. Solvent-cast PLGA films were aminolyzed and modified by a Layer-by-Layer technique to obtain a nano-layered coating with poly(sodium4-styrenesulfonate) and poly(allylamine hydrochloride) as polyelectrolytes [71]. A water-soluble antibiotic, metronidazole (MET), was incorporated to successfully endow the film with excellent antibacterial properties against the keystone periodontal pathogen *P. gingivalis*, without compromising the in vitro biocompatibility.

Recently, intensive efforts are being made all over the world to improve the effectiveness of delivery systems. Due to the rapid advancement in nanotechnology, nanoparticulate systems provide several advantages as compared with other delivery systems. A nanoparticle is defined as a particle that has a size range of approximately 1–100 nm, with properties that are not shared by non-nanoscale particles with the same chemical composition. Regarding the application in periodontitis, first, it is easier for nanoparticle carriers to access deeper into the site of tooth furcation and periodontal pocket regions under gum line, while it may be inaccessible for other delivery systems [21]. Second, frequency reduction of administration in the periodontal pockets by applying nanoparticles would enhance the therapeutic effects and reduce side effects [7]. Third, nanoparticles possess better stability in biological fluid and high dispersibility in an aqueous medium [7]. Sadat et al. prepared both PLGA and PEGylated PLGA nanoparticles by various methods, such as single and double solvent evaporation emulsion, ion pairing, and nanoprecipitation [67]. Antibacterial properties against *Aggregatibacter actinomycetemcomitans* (*A. actinomycetemcomitans*) showed that the encapsulation of minocyline into the polymeric nanoparticles improved the antibacterial efficiency by two folds, compared to that of the free drugs. This was possibly due to better penetration of the nanoparticles into bacterial cells and better delivery of minocyline to the site of action [67]. Besides encapsulation, drugs can also be doped onto the surface of polymeric nanoparticles due to the specific surface chemistry. A novel type of polymeric nanoparticles, PolymP-*n* active nanoparticles, was recently developed, with potential for periodontal regeneration [72]. Due to the surface chemistry of the nanoparticles, containing functional groups with sequences of anionic carboxylate (i.e. COO^−^), it was possible to dope metal cations (in this case calcium, zinc and silver) and antibiotics (doxycycline), with potential antibacterial activity [73]. A multispecies periodontal biofilm was developed by using *Streptococcus oralis* (*S. oralis*), *Actinomyces naeslundii* (*A. naeslundii*), *Veillonella parvula* (*V. parvula*), *Fusobacterium nucleatum* (*F. nucleatum*), *P. gingivalis* and *A. actinomycetemcomitans* to investigate the antibacterial properties of polymeric PolymP-n active nanoparticles doped with different substances: zinc, calcium, silver and doxycycline [73]. A similar biofilm formation was observed, although reductions in bacterial viability were detected in biofilms in contact with the different nanoparticles, and were more pronounced with silver and doxycycline nanoparticles. PolymP-n nanoparticles with doxycycline resulted in unstructured biofilm formation and significantly lower colony units of the six species, compared with the other specimens and controls [73]. However, with the emergence and increase of microbial resistance to antibiotics, further studies should investigate not only antibiotic-free delivery systems but also antimicrobial peptides (AMPs) for treating periodontal infections. Bacterial flora is controlled initially by the innate immune system of oral epithelia, saliva and gingival crevicular fluid, which are rich with AMPs [74]. AMPs such as LL37 and β-defensins have demonstrated excellent antibacterial efficacy against periodontal pathogens [75]. Therefore, the existing and newly-identified AMPs may be promising for therapeutic uses in treating periodontal disease, and they may serve as templates for peptides and peptide-mimetics with improved therapeutic efficacy [74].

## 3. Antibacterial Polymeric Materials Against Periodontal Pathogens

Although polymers can serve as matrix materials holding the antibacterial agents for treatment of infectious disease, the development of polymers with antimicrobial activity themselves is also an important area of research. In addition, increasing antibiotic drug-resistance of microorganisms has drawn considerable attention toward the development of new types of antibacterial agents. Several antibacterial polymers are applied in infectious diseases caused by pathogenic microorganisms. However, their antibacterial properties against periodontal pathogens or biofilms are rarely investigated [9,76]. 

Chitosan, a linear polycationic hetero polysaccharide copolymer, exhibits an excellent capacity of antimicrobial efficacy. The contact between negatively charged cell wall and positively charged chitosan can alter the cell wall permeability and eventually lead to the complete cell wall disruption and cell death. Molecular weight, concentration, and hydrophilic/hydrophobic characteristics of chitosan also play some role in antibacterial efficacy [77]. Chitosan showed antimicrobial activity against periodontal pathogens *P. gingivalis, A. actinomycetemcomitans* and *Prevotella intermedia* (*P. intermedia*) with quick and efficient bactericidal activity [78]. Similar excellent antibacterial activity of chitosan against *P. gingivalis* and *A. actinomycetemcomitans* was also reported by Arancibia et al. [79]. Furthermore, Sarasam et al. confirmed that chitosan-mediated antibacterial activity was contact-dependent; therefore, blending chitosan with other components such as polycaprolactone (PCL) compromised its antibacterial activity against *A. actinomycetemcomitans* [80]. The possible explanation is that the surface characteristics of chitosan, such as surface roughness and charge distribution, may be altered when blending with PCL, thereby decreasing the antibiotic performance. 

**Figure 1:**
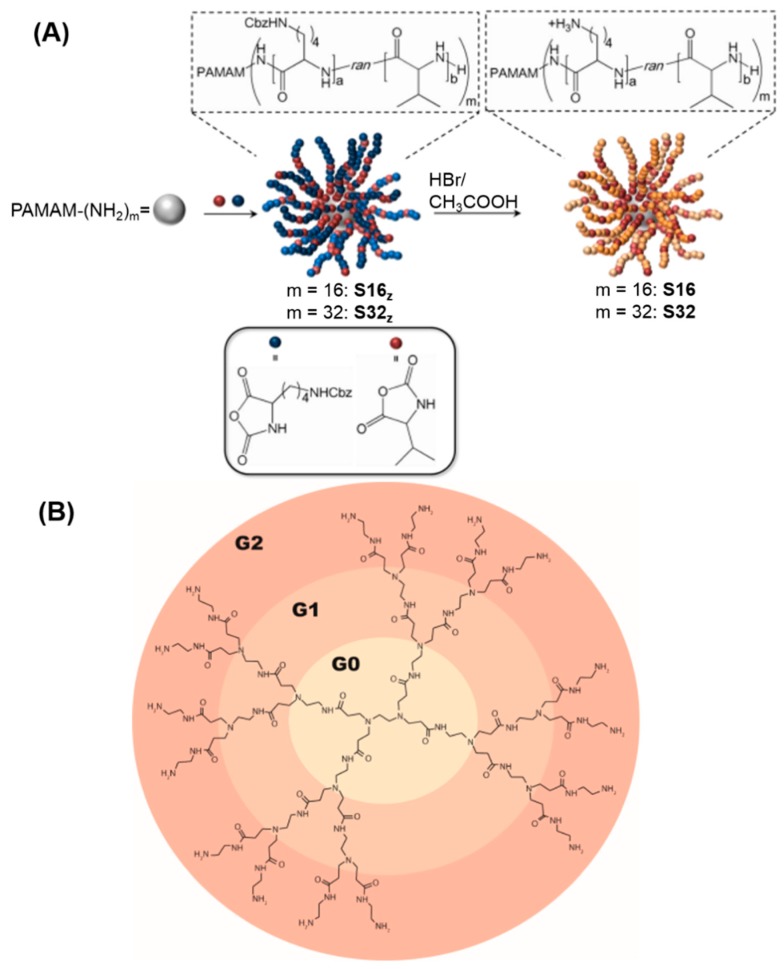
Synthesis of SNAPPs. (**A**) Synthesis of SNAPPs via ring-opening polymerization of lysine and valine N-carboxyanhydrides (NCAs) was initiated from the terminal amines of poly(amido amine) (PAMAM) dendrimers. Second- and third-generation PAMAM dendrimers in (**B**) with 16 and 32 peripheral primary amines were used to prepare 16- and 32-arm SNAPPs, respectively. Note that the number of initiating points on the figure does not reflect the actual number, which is 16 or 32. The number of repeat units for lysine and valine are a and b, respectively. (Reproduced with permission from [81]. Spinger Nature, 2016.)

Recent efforts developed a new class of antimicrobial agents, termed “structurally nanoengineered antimicrobial peptide polymers” (SNAPPs), as shown in Figure 1. They exhibited sub-μM activity against Gram-negative bacteria *Acinetobacter baumannii* (*A. baumannii*) [81]. It is possible that in addition to putative periodontal pathogens, non-oral bacterial species such as *A. baumannii* may also play a role in the etiopathogenesis of periodontal diseases [82]. The antibacterial activity of SNAPPs proceeds via a multimodal mechanism of bacterial cell death by outer membrane destabilization, unregulated ion movement across the cytoplasmic membrane and induction of the apoptotic-like death pathway [81]. Therefore, SNAPPs showed great promise as low-cost and effective antimicrobial agents in combating the growing threat of Gram-negative bacteria, which is prevalent in periodontitis.

## 4. Antibacterial Polymeric Membrane for GTR Inhibiting Periodontitis 

GTR strategies are widely applied for periodontal tissue regeneration. Generally, a mechanical barrier membrane is established to create a protected space over bone defect and prevent the apical migration of the gingival epithelium. This facilitates the growth of periodontal ligament and bone tissue to selectively re-attach the root surface [12]. To enhance the bioactivity and adjust the degradation rates, polymeric materials such as collagen, chitosan, gelatin, silk fibroin, and synthetic polymers (PLGA, PCL and poly (ethylene glycol) (PEG)) are used to fabricate the GTR membranes. 

Clinically, infection is a major reason for GTR failure [83]. Infection can be caused by either pathogen colonization at the wound site or foreign body response to the implant material [84]. Periodontal disease and GTR implant-related infections mainly result from anaerobic bacteria such as bacteroides species, fusobacteria, and clostridia. Hence, multifunctional GTR membranes were recently developed to not only possess barrier and tissue regenerative properties, but also exhibit antibacterial effects against periodontal pathogens. 

Metronidazole (MET) is widely used to treat periodontitis in patients for whom mechanical debridement is not successful or possible [85]. Xue et al. developed MET-loaded electrospun PCL nanofiber membranes. A wide range (1–40 wt %) of MET was incorporated into the membrane. An in vitro antibacterial effect against *F. nucleatum* showed clear inhibition zones around the membranes. GTR membranes with 30 wt % MET showed excellent comprehensive properties. However, the release time of metronidazole was only a few days [86]. To sustain the drug release for a longer time, gelatin was added into PCL in the same electrospun system to form a PCL/gelatin nanofiber membrane [87]. As effective MET carriers, PCL/gelatin membranes with various contents of MNA showed an approximately 60% release of MET within one week. This was followed by a sustained release of up to three weeks, which was predominantly controlled by diffusion and gelatin degradation [87]. In addition, an efficient anti-infective GTR membrane was developed by doping drug-loaded clay nanotube into electrospun PCL/gelatin microfibers [88]. By combining the drug-loading capability of nanotubes and direct drug loading into electrospun microfiber matrix, composite membranes with a three-week sustained drug release capability were realized. This extended release prevents the colonization of *F. nucleatum* over period of three weeks [88]. This three-week prolonged drug release is considered to be an optimal treatment time period. 

**Figure 2:**
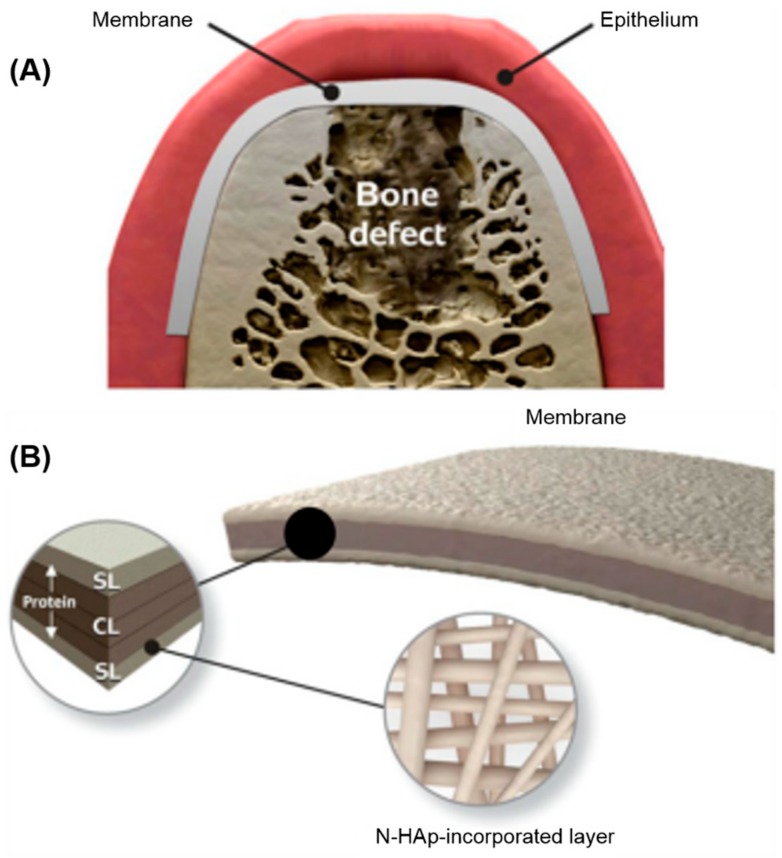
Schematic illustration of the spatially designed and functionally graded periodontal membrane. (**A**) Membrane placed in a guided bone regeneration scenario. (**B**) Details of the core layer (CL) and the functional surface layers (SLs) interfacing bone (n-HAp) and epithelial (MET) tissues. Note the chemical composition step-wise grading from the CL to SLs, i.e., polymer content decreased and protein content increased. (Reproduced with permission from [89]. Elsevier, 2011).

Bittino et al. fabricated a novel functionally-graded periodontal membrane containing MET with a spatially designed layer structure via sequential electrospinning [89]. As shown in Figure 2, the functionally-graded periodontal membrane consisted of a core layer (CL) and two functional surface layers (SLs) interfacing with bone (nano-hydroxyapatite, n-HAp) and epithelial (MET) tissues. This multi-layered electrospun GTR membranes had nano-sized hydroxyapatite for osteoconductive/inductive behavior, and metronidazole to combat periodontal pathogens. Other antibiotics such as amoxicillin, tetracycline, chlorhexidine and doxycycline were also incorporated into polymeric membrane with therapeutic properties [90,91,92,93]. Composite GTR membranes with polytetrafluoroethylene (ePTFE) and collagen were loaded with chlorhexidine and inhibited *A. actinomycetemcomitans*, allowing the attachment of periodontal ligament cells [92]. Similar results were obtained when amoxicillin and tetracycline were incorporated into GTR membranes [94].

Besides antibiotic addition, metal or metallic oxide such as silver, zinc, copper and zinc oxide nanoparticles were also incorporated into the GTR membranes. For example, zinc oxide (ZnO) nanoparticles not only introduced an antibacterial activity, but also improved the osteoconductivity of the periodontal membrane [12]. Nasajpour et al. developed a GTR membrane through electrospinning methods with a dispersed solution of ZnO within a polymeric carrier PCL. Incorporation of 0.5% (*w*/*v*) ZnO nanoparticles provided antibacterial effects against periodontal pathogens *P. gingivalis*, while supporting the viability and the osteodifferentiation of the seeded periodontal ligament stem cells, without negatively impacting their biocompatibility feature [12]. Zinc phosphate was also loaded into the GTR membrane by an immersion method. The zinc phosphate mineralized membranes possessed a strong antibacterial effect against *A. actinomycetemcomitans* [95]. A recent study developed a chitosan/polyurethane (CSP) nanofibrous membrane with silver nanoparticles (AgNPs) in the membrane [96]. The antibacterial effects of the membrane against *P. gingivalis* was maintained when the AgNP level was adjusted to be at a nontoxic level.

It was reported that high drug ionic strength and rapid solvent evaporation favored the presence of the drug on the membrane surface, which led to a high initial burst release, thereby avoiding and/or eliminating biofilm formation [97]. However, the high burst release and short release period could not effectively prevent bacterial infections. Hence, it is highly desirable to develop novel GTR membranes with sustained and controlled release of antibacterial agents, particularly for patients with a predisposition to such complications: smokers, patients with diabetes mellitus, etc. [98].

## 5. Antibacterial Polymeric Composites to Combat Periodontal Pathogens

To suppress oral biofilm/plaque buildup and increase the restoration’s longevity, non-agent-leaching monomers are incorporated into dental polymers. Imazato et al. combined alkylpyridinium, a type of quaternary ammonium methacrylates (QAM), with a methacrylate, and synthesized a novel monomer, 12-methacryloyloxydodecylpyridinium bromide (MDPB) [99]. The QAM was chemically copolymerized with the resin by forming a covalent bonding with the polymer network [100,101]. While the quaternary ammonium group was responsible for the antibacterial activity of QAM, the methacrylate group allowed for the copolymerization with other conventional monomers. Therefore, the antibacterial QAM immobilized in the resin matrix was not released or lost over time, thus providing a durable antibacterial capability. The three likely mechanisms for bactericidal QAMs against microorganism were: (1) contact between negatively charged bacteria and positively charged QAMs leading to osmotic pressure; (2) diffusion through the cell wall and binding to the cytoplasmic membrane; and (3) disruption of the cytoplasmic membrane, releasing of cytoplasmic constituents, and cell death [102,103,104]. Several papers review the antibacterial monomers in dental resins [105,106,107,108], as well as studies on antibacterial functions against cariogenic bacteria and oral biofilms to inhibit dental caries [109,110,111,112]. Therefore, the present article focuses on new developments in antibacterial dental resins for root caries restorations with an emphasis on their antibacterial effects on periodontal pathogens.

Elderly people suffer more risks of root caries because of gingival recession and less saliva flow [113]. Periodontitis-related gingival recession often leads to more exposure of the root surfaces, which results in the increased risk of root caries. Root surfaces differ from enamel surfaces with a lower mineral content and a higher amount of organic materials [114]. Therefore, root caries lesions may occur on all exposed root surfaces but are mainly found in biofilm retention sites [114]. Root caries can be treated with a Class-V restoration, whose margins are often subgingival, which can hinder cleaning and provide pockets for bacterial growth, thus gradually resulting in the loss of the periodontal attachment of the tooth. Indeed, it is well-established that microbial biofilms are the primary etiological factor that causes periodontitis [5]. Recent research demonstrated that *P. gingivalis*, *P. intermedia* and *A. actinomycetemcomitans* are the three major bacterial species that contribute to periodontitis and peri-implantitis in subgingival plaque [115]. In the periodontal pockets, these bacteria can generate virulence factors that lead to the gradual loss of the supporting bone [115]. In addition, *Prevotella nigrescens* (*P. nigrescens*) is associated with both healthy and diseased conditions of the periodontium [116]. *F. nucleatum* is correlated with the progress in periodontitis [117] and can enhance the invasion into human gingival epithelial and endothelial cells by *P. gingivalis* [118]. Furthermore, *Enterococcus faecalis* (*E. faecalis*) is regarded as an endodontic pathogen and also detected in subgingival plaque and saliva of patients with chronic periodontal disease [119]. 

**Figure 3:**
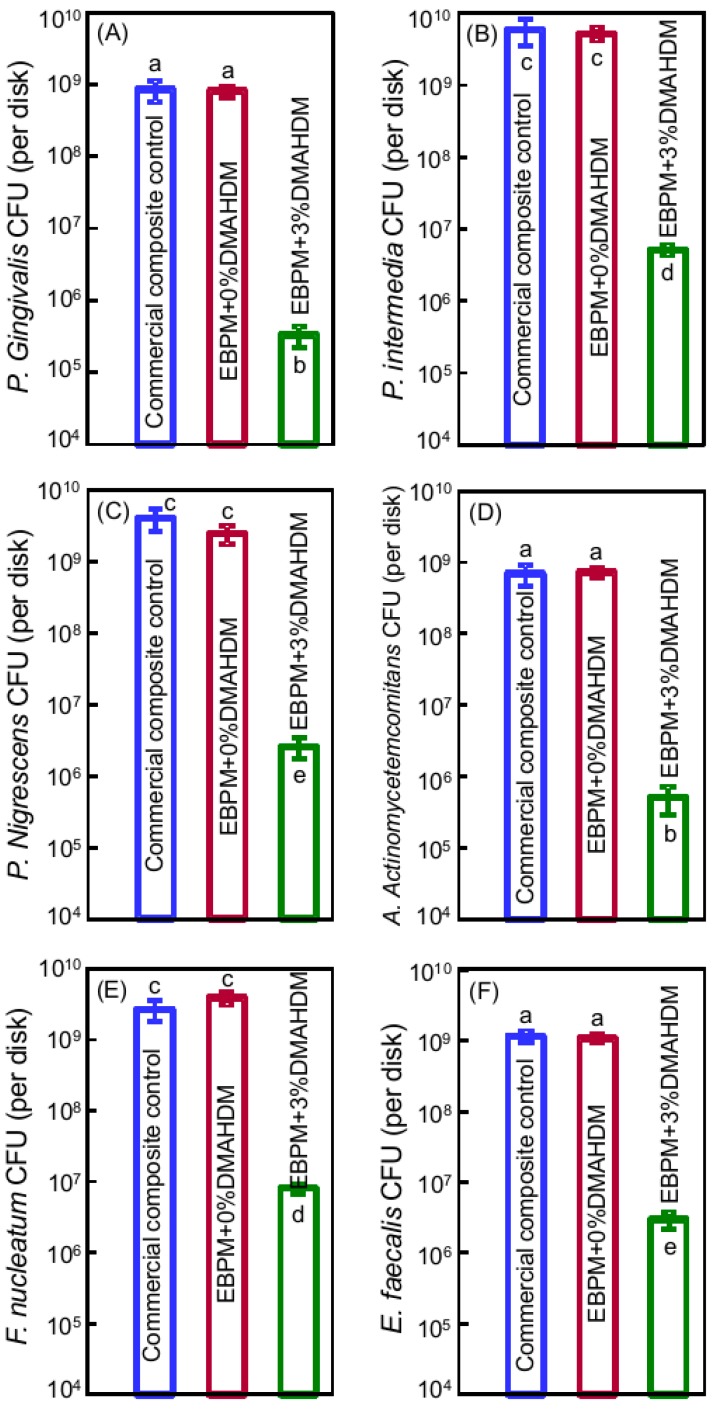
CFU counts of two-day biofilms on composites (mean ± SD; *n* = 6): (**A**) *P. gingivalis*; (**B**) *P. intermedia*; (**C**) *P. nigrescens*; (**D**) *A. actinomycetemcomitans*; (**E**) *F. nucleatum*; and (**F**) *E. faecalis*. Note the log scale for the y-axis. CFU counts on composite containing DMAHDM were nearly three orders of magnitude lower than composite without DMAHDM. Bars with dissimilar letters are significantly different from each other (*p* < 0.05). (Reproduced with permission from [120]. Elsevier, 2016.)

Therefore, these six species were selected in a recent study [120]. A polymeric composite for Class-V tooth cavity restorations was developed with therapeutic functions to combat these six species of pathogens related to the start and the exacerbation of periodontitis [120]. The polymeric matrix in the composite was composed of ethoxylated bisphenol A dimethacrylate (EBPADMA) and pyromellitic dianhydride glycerol dimethacrylate (PMGDM) at a 1:1 mass ratio (referred to as EBPM). A novel antibacterial monomer dimethylaminohexadecyl methacrylate (DMAHDM) was added at a mass ratio of 3%. DMAHDM incorporation did not compromise the mechanical properties of polymeric composite. The flexural strength of EBPM + 3% DMAHDM was similar to that of a commercial control composite that had no antibacterial effect [120]. Static single-species biofilms of six types of pathogens were used to evaluate the antibacterial effects of polymeric composites with DMAHDM incorporation. For all six species, incorporating 3% DMAHDM into EBPM composite decreased the biofilm CFU by several orders of magnitude, compared to the commercial control and the 0% DMAHDM group (*p* < 0.05). DMAHDM composite reduced the CFU of different bacterial species differently, some by slightly less than 3 log, others by more than 3 log (as shown in Figure 3) [120]. The killing efficacy of DMAHDM composite against the six species was: *E. faecalis* < *F. nucleatum < P. nigrescens = P. intermedia* < *A. actinomycetemcomitans* < *P. gingivalis*. Furthermore, the biomass and the polysaccharide production by biofilms were also reduced on the EBPM composite with 3% DMAHDM, compared to control composite [120]. 

**Figure 4:**
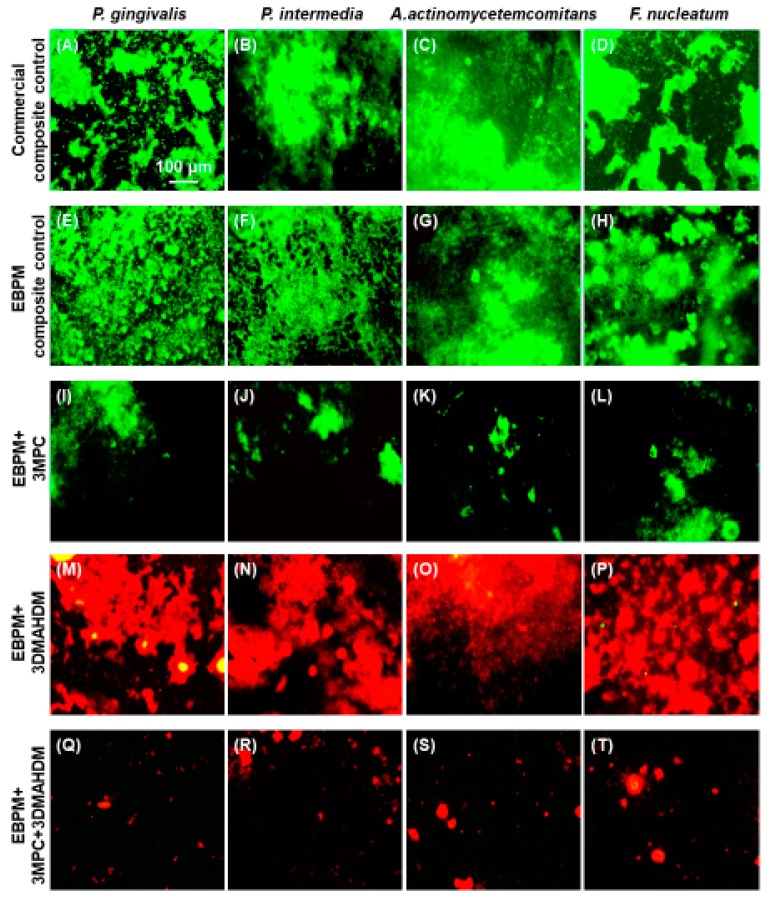
Representative live/dead staining images of two-day biofilms of four species of periodontal pathogens on the five composites: (**A**–**D**) Commercial composite control; (**E**–**H**) EBPM composite control; (**I**–**L**) EBPM + 3MPC; (**M**–**P**) EBPM + 3DMAHDM; and (**Q**–**T**) EBPM + 3DMAHDM + 3MPC. All images have the same scale bar as shown in (**A**). Live bacteria were stained green. Dead bacteria were stained red. Composites without DMAHDM had primarily live bacteria. EBPM + 3DMAHDM + 3MPC had much less bacterial adhesion, and the biofilms consisted of primarily dead bacteria. DMAHDM: dimethylaminohexadecyl methacrylate; MPC: 2-methacryloyloxyethyl phosphorylcholine (Reproduced with permission from [127]. Elsevier, 2016.)

Salivary protein adsorption on the surface is required for pellicle formation and is a prerequisite for oral bacteria adherence [121]. Based on this mechanism, a protein-repellent composite was developed using 2-methacryloyloxyethyl phosphorylcholine (MPC). MPC is a methacrylate with a phospholipid polar group in the side chain [122]. MPC has strong protein-repellency, and has been incorporated into artificial blood vessels, hip joints, and microfluidic devices [123,124,125]. Several MPC-containing medical devices have won the approvals of the United States Food and Drug Administration, and have been used clinically [123,126]. A recent study developed a multifunctional polymeric dental composite containing bioactive agents (MPC for protein-repellency, and DMAHDM for anti-biofilm activity) to suppress periodontal pathogens [127]. These results showed that adding up to 3% MPC and 3% DMAHDM did not compromise the strength and elastic modulus, compared to the control. Representative live/dead images of two-day biofilms for the 5 × 4 full-factorial design are shown in Figure 4. Live bacteria were stained green, and bacteria with compromised membranes were stained red. For all four species periodontal biofilms (*P. gingivalis*, *P. intermedia*, *A. actinomycetemcomitans*, and *F. nucleatum*), the two control composites were covered by live bacteria. In contrast, composite with 3% MPC had much less bacterial adhesion. Composite with 3% DMAHDM had substantial dead bacteria. Composite with 3% DMAHDM + 3% MPC had much less bacterial adhesion, and the bacteria were mostly dead [127]. Dual agents of MPC + DMAHDM incorporation into polymeric composites exerted a much greater anti-biofilm activity against periodontal pathogens, than using MPC or DMAHDM alone. Therefore, the polymeric composite containing 3% DMAHDM and 3% MPC appeared to be the optimal composition. It showed a high potential for applications in Class-V restorations to inhibit periodontal biofilms, by reducing biofilm CFU by nearly four orders of magnitude for all the periodontitis-related pathogens examined in that study [127].

Previous studies tested single species biofilms [128]. However, single species models are not representative of natural biofilms where multispecies communities are by far the most predominant [129]. The eradication of multispecies biofilms is more difficult to achieve than single species, as multispecies biofilms are more highly resistant to antimicrobial agents than single species [130]. Furthermore, the biofilm composition may influence the outcome of periodontitis treatments [131] and the killing efficacy of antibacterial agents [130]. 

**Figure 5:**
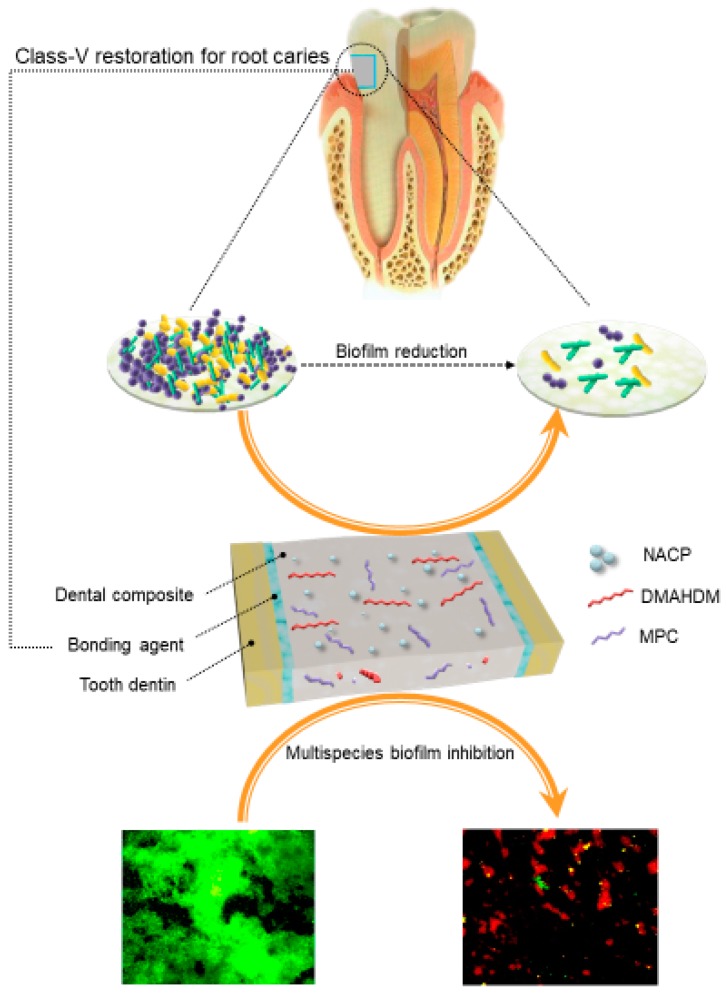
Antibacterial strategy using dual agents in dental composite. Dimethylaminohexadecyl methacrylate (DMAHDM) can inactivate periodontal pathogens by contact without leaching from resins. Methacryloyloxyethyl phosphorylcholine (MPC) can detach proteins, thereby hampering bacterial attachment. DMAHDM and MPC are both non-volatile, chemically stable and can sustain long-term antibacterial activity. DMAHDM: dimethylaminohexadecyl methacrylate; MPC: 2-methacryloyloxyethyl phosphorylcholine; NACP: nanoparticles of amorphous calcium phosphate (Revised and resubmitted to Dent. Mater. Ref [132].)

Therefore, a recent study investigated the effects of the number of species (from 1 to 9) in the periodontal biofilm on the inhibition efficacy of the composite [132]. Moreover, the effect of dual agents (MPC + DMAHDM) versus single agent on the inhibition efficacy was investigated as a function of the number of species in the biofilm for the first time. Figure 5 illustrates the anti-biofilm strategy. The bioactive composite reduced protein adsorption by an order of magnitude and greatly reduced biofilm viability. It decreased the biofilm CFU by more than three orders of magnitude for all four types of periodontal biofilms (single-species, three-species, six-species, and nine-species biofilms), compared to the control composite. With increasing the biofilm species from 1 to 9, the antibacterial efficacy of DMAHDM composite decreased; the folds of CFU reduction decreased from 947 to 44 folds. In contrast, the DMAHDM+MPC composite maintained a CFU reduction folds of greater than 3000, showing a similarly high antibacterial potency from 1 to 9 species in the biofilms. 

Therefore, DMAHDM composite was more effective in inhibiting single species biofilm; the inhibition efficacy decreased with increasing the number of species in the biofilms. Adding MPC into the DMAHDM composite increased the efficacy against multi-species biofilms, achieving nearly the same high efficacy against biofilms with 1–9 species. The novel nanocomposite containing dual agents (MPC + DMAHDM) with potent antibiofilm and protein-repellent functions is promising for Class-V restorations to treat root caries, inhibit periodontal pathogens, and protect the periodontal tissues.

To enhance the antibacterial efficacy, Xiao et al. incorporated nanoparticles of silver (NAg) into the aforementioned MPC + DMAHDM composites to inhibit pathogens such as *A. actinomycetemcomitans*, *F. nucleatum* and *P. gingivalis* [133]. There are several advantages for NAg incorporation into polymeric nanocomposite. (1) The small particle size of NAg (2.6 nm) yielded a relative high surface area to mass ratio, which enabled a small quantity of NAg to be sufficient for the composite to be strongly antibacterial. This avoided the need to use a high mass fraction of NAg and compromised the nanocomposite’s mechanical properties and esthetics [134]. (2) Ag is known to have a superior biocompatibility and low toxicity to humans. (3) Resins containing NAg exhibited long-lasting antimicrobial properties. For example, a previous study demonstrated that NAg-containing resins showed antibacterial activity even after 12 months of water-aging [135]. (4) Ag have less possibility for causing bacterial resistance than antibiotics, which alleviates the drug-resistance concern [136]. (5) NAg is capable of long-distance killing of bacteria due to the release and diffusion of the Ag ions. This is expected to be useful to inhibit the suspended bacteria in the periodontal pockets away from the composite surface. Indeed, the combination of MPC, DMAHDM and NAg achieved the reduction in biofilm CFU by nearly five orders of magnitude, which is much greater than those achieved by previous antibacterial dental composites [120,127].

Glass ionomer materials have been widely used in Class-V restorations clinically due to their fluoride release, good biocompatibility with dental pulp tissues, and ability to bond to tooth structures. Fluoride release has a slight antimicrobial effect and a good remineralization effect. However, a previous study indicated that a commercial resin-modified glass ionomer cement was not potent enough to inhibit bacterial growth and biofilm formation [137]. In addition, for anterior teeth, cervical lesions need to be esthetic, which can be better satisfied with a resin composite. Therefore, the aforementioned polymeric dental composites are promising for Class-V restorations due to their strong antibacterial effects against periodontal pathogens.

## 6. Polymeric Bonding Agent Inhibiting Periodontal Pathogens

A dental bonding agent is used to bond restorations to teeth. A previous study developed a polymeric bonding agent with nanoparticles of amorphous calcium phosphate (NACP) and DMAHDM for tooth root caries restorations and endodontic applications [138]. A primer contained PMGDM and 2-hydroxyethyl methacrylate (HEMA) at a mass ratio 10:3, with 50% acetone solvent (all by mass ratio). The adhesive contained PMGDM, EBPADMA, 2-hydroxyethyl methacrylate (HEMA) and BisGMA at 45/40/10/5 mass ratio (referred to as PEHB) [135]. *P. gingivalis*, *P. intermedia*, *P. nigrescens*, *A. actinomycetemcomitans*, *F. nucleatum* and *Parvimonas micra* (*P. micra*) were used to represent periodontal pathogens, and *Enterococcus faecium* (*E. faecium*) and *E. faecalis* were selected as endodontic pathogens. Therefore, eight types of single-species biofilms were formed on resins. Adding 5% DMAHDM and 30% NACP into the adhesive resin did not negatively affect the dentin bond strength. Biofilm CFU was reduced by nearly three orders of magnitude via the therapeutic resin. The inhibition efficacy via DMAHDM-containing resin was ranked as: *P. gingivalis* > *A. actinomycetemcomitans* > *P. intermedia* > *P. nigrescens* > *F. nucleatum* > *P. micra* > *E. faecalis* > *E. faecium*. Biofilm biomass, metabolic activity and polysaccharide were also greatly reduced via DMAHDM [138].

In addition, three bioactive agents (NACP for remineralization, MPC for protein-repellency, and DMAHDM for anti-biofilm activity) were combined into a polymeric bonding agent (PEHB) to suppress periodontal pathogens [139]. PEHB with 5% DMAHDM showed a strong antibacterial function and protein-repellent properties. The use of dual agents, 5% DMAHDM + 5% MPC, achieved a greater killing efficacy against *P. gingivalis* single-species biofilm than against multi-species biofilm. However, this novel polymeric bonding agents still showed great reduction in multispecies biofilm growth, metabolic activity and polysaccharide production, yielding three orders of magnitude in CFU reduction [139].

The mechanism of contact-inhibition implied that the polymer surface was separated from the overlaying biofilm by the presence of the salivary protein pellicles on the polymer surface. This would reduce the extent of contact, and hence result in the decreased contact-inhibition efficacy. Therefore, because of the protein-repellency of the MPC, it helped diminish protein coverage on the polymer surface, thus exposing more polymer surface with quaternary amine N^+^ sites, thereby promoting the contact-inhibition ability. Therefore, the dual use of DMAHDM and MPC in the dental polymer could work synergistically to maximize the periodontal bacteria inhibition capability.

Besides the antibacterial agents, NACP were also incorporated in polymeric bonding agents [140,141,142]. While having little antibacterial activity, the NACP composite was “smart” and could greatly increase the Ca and P ion release at a cariogenic low pH, when such ions were most needed to inhibit caries [143]. Indeed, a previous study showed that an NACP nanocomposite successfully remineralized enamel lesions, and achieved an enamel lesion remineralization efficacy that was four-fold greater than that of a commercial fluoride-releasing control [144]. In addition, a previous study showed that NACP composite could be recharged repeatedly with Ca and P ions, which ensured that it could continuously release Ca and P ions to provide long-term remineralization [145]. In Class-V restorations, these Ca and P ions from the composite are expected to help remineralize tooth roots, reduce root sensitivity, neutralize biofilm acids, and protect the root structures.

Currently, several contemporary dental adhesives have been reported to possess a favorable “immediate” bond strength to enamel and dentin. However, the clinical longevity of the bonded restorations is still too short, mainly due to the degradation of the adhesive tooth-composite interface, especially for Class-V restorations with subgingival margins. First, in comparison with bonding composite resins to enamel, bonding to cervical dentin is less predictable due to the relatively low density and oblique orientation of dentinal tubules in cervical dentin in Class-V restoration [146]. Second, marginal openings are located most frequently at the dentin/cementum margins when Class-V cavities are restored with composite-based materials. Besides polymerization stress, forces that result from volumetric changes due to temperature fluctuations and non-uniform deformation of restoratives and the tooth substance during functional loading also repeatedly stress the restorative interface [147]. Third, the subgingival cavity is difficult to dry. The inadequate air drying could result in too much residual solvent in the adhesive and in the hybrid layer, which would reduce the bonding durability [148]. Therefore, advanced restorative techniques such as the incremental filling technique with the application of flowable materials as an intermediate layer are used to improve the marginal adaptation of composites to the dentin/cementum margin in Class-V restoration [147].

Investigation of the toxicity of the antimicrobial monomers is a prerequisite before their use in oral restorations. Imazato et al. indicated that the first QAM for dental application, MDPB, exhibited a low level of toxicity to human pulpal cells, similar to the diluent resin monomer triethyleneglycol dimethacrylate (TEGDMA) [101]. Although a previous study demonstrated that the increasing monomer concentration and increasing chain length of QAMs would increase the cytotoxicity, QAMs with chain lengths from 3 to 18 were shown to have less cytotoxicity than BisGMA [149]. At monomer concentrations of ≤2 µg/mL, all the tested QAMs with CL ≤ 16 had cytotoxicity matching that of HEMA and TEGDMA [149]. Clinically, a typical tooth cavity would use approximately 20 µg of the bonding agent. In the unrealistic worst-case scenario, assume that all 20 µg were leached out in one day [149]. Human saliva flow is approximately 1000–1500 mL/day [150]. This would yield a monomer concentration of 0.02 µg/mL, which would be acceptable for clinical applications.

## 7. Polymeric Materials for Antibacterial Photodynamic Therapy Against Periodontal Pathogens

**Figure 6:**
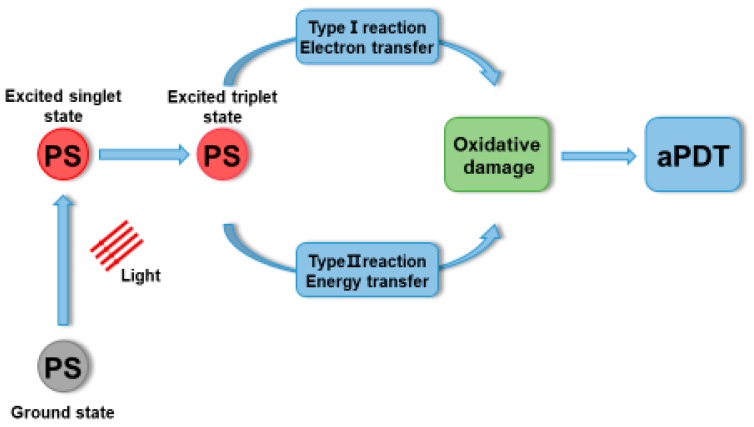
Schematic representation of mechanism of aPDT. Triggered by the light, ground state photosensitizer transfer into excited singlet state and triplet state. The triplet state can undergo type I (electron transfer) reaction and type II (energy transfer) reaction to produce singlet oxygen, which can cause oxidative damage.

Currently, mechanical debridement and antibiotic therapy are still the major approaches for periodontitis treatment. However, conventional tools can hardly reach deep periodontal pockets. The overuse of antibiotics could lead to drug-resistant bacteria. In the past decade, antibacterial photodynamic therapy (aPDT) gradually attracted the attention of researchers, and several studies on the efficient clearance of periodontal pathogens or biofilms using aPDT were reported [151,152]. aPDT is a promising antibacterial therapeutic approach for periodontal pathogens to make up for the aforementioned shortcomings. aPDT involves three components: photosensitizer (PS), light, and oxygen. For periodontal pathogens, PS should have properties of good biocompatibility, high selectivity, high solubility, light stability, high quantum yield of reactive oxygen species (ROS) generation having positive charge, and causing no staining on gingiva [153,154]. The ideal irradiation light should be at red to near-infrared wavelengths to obtain a deeper penetration. As shown in Figure 6, the mechanism of aPDT is that, after being excited by a specific wavelength of light, the PS converts energy or electrons to generate ROS in the presence of molecular ground triplet state oxygen, thus leading to bacterial cell death [155]. Generally, the bactericidal effect of aPDT can be attributed to oxidative damage, including DNA damage and cell membrane system damage (biofilm matrix destruction, lipid oxidation, cell surface damage, etc.) [156].

Recently, attention has been paid to biodegradable polymeric materials in PDT studies due to their most remarkable properties of biocompatibility and low toxicity [157]. Regarding aPDT, application of polymeric materials carries several advantages: (1) increasing positive charge of PS and enhancing the binding between PS and bacteria; (2) reducing aggregation of PS and increasing its bioavailability; and (3) serving as a carrier or scaffold with good biocompatibility and biodegradability.

Periodontitis is a chronic infectious disease associated with Gram-negative bacteria. The cell envelope of Gram-negative bacteria consists of an outer membrane, a thinner peptidoglycan layer and a cytoplasmic membrane. Lipopolysaccharides (LPS) and the porins, the two main components of the outer membrane, have the membrane barrier protection to allow only certain molecules to pass through [158]. Negatively charged LPS molecules have a strong affinity for cations or cationic group. In contrast, anionic and neutral group cannot be up-taken by bacteria, which hinders the effective implementation of aPDT [159]. Thus, cationic PSs are considered to be used to overcome the initial difficulty in aPDT. 

**Figure 7:**
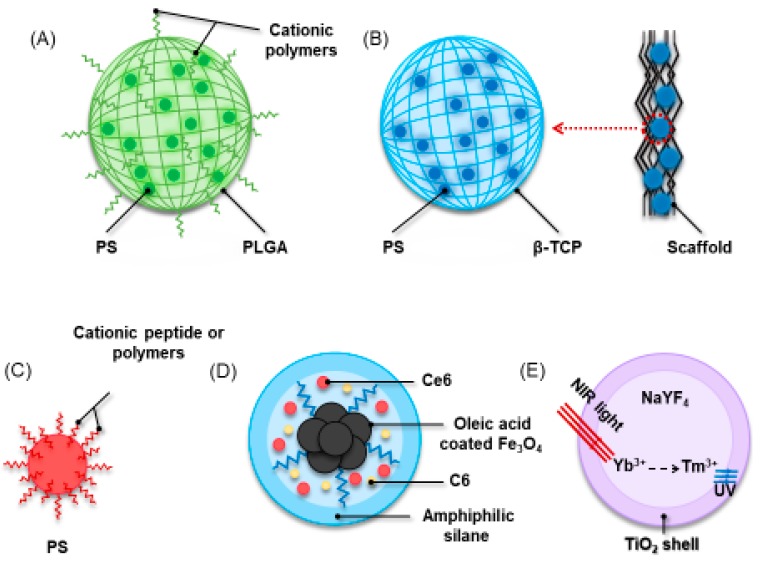
The various strategy of photosensitizer (PS) modification by polymeric materials to enhance bactericidal effect: (**A**) PS-loaded PLGA nanospheres with cationic polymers; (**B**) a scaffold or a carrier for PS; (**C**) PS binding to cationic peptide or polymers; (**D**) the structure of antibacterial multifunctional nanoparticles Fe_3_O_4_-silane@Ce6/C6; and (**E**) the structure of nanoparticles NaYF_4_:Yb^3+^, Tm^3+^@TiO_2_.

Figure 7 depicts different strategies of PS modification by polymers for bactericidal effect enhancement. In a recent study, compared with free methylene blue (MB) alone, MB-loaded PLGA cationic nanoparticles showed greater bactericidal effect on microorganisms in both planktonic and biofilm phase from human dental plaque samples collected from untreated patients with chronic periodontitis (Figure 7A) [160]. With a concentration of 50 mg/mL, exposure to red light for 5 min with a power density of 100 mW/cm^2^, MB-loaded PLGA cationic nanoparticles reduced bacterial viability by approximately 60% from the planktonic PDT and 48% from the biofilm PDT [160]. In addition, the effect of this MB-NP-based aPDT as an adjunctive treatment combining with ultrasonic scaling (US) and scaling and root planning (SRP) showed a slightly better outcome than using US + SRP alone [161]. All clinical parameters including visible plaque and gingival bleeding indexes (GBI), bleeding on probing and probing pocket depth in both groups showed the greatest improvement one month later. However, US + SRP + aPDT showed a greater effect (28.82%) on GBI compared with US + SRP at three months [161]. Similarly, indocyanine green-loaded nanospheres coated with chitosan (ICG-Nano/c) was designed to cope with the clearance of *P. gingivalis.* ICG-Nano/c could adhere to the surface of *P. gingivalis* and significantly reduced the number of *P. gingivalis* by an order of 2 log_10_(96.71%) to 4 log_10_(99.99%) [162,163]. In addition, the strategy of intermittent irradiation with air cooling also improved therapeutic penetration and prevented tissue thermal damage [162]. This was probably because the PLGA provided positive charge and helped aPDT to produce a sustained effect on the periodontal lesions for a long time [164]. 

Recently, a chitosan-based hydrogel containing 0.5% hydroxypropyl methylcellulose and toluidine blue O with high adhesiveness was developed for the treatment of periodontitis [165]. Two major periodontal pathogens, *A. actinomycetemcomitans* and *P. gingivalis* biofilms, were used to evaluate the bactericidal effects. When the irradiation power was higher than 32.4 J·cm^−2^ using 630 nm light, a significant reduction in survival rate was observed in both single-species periodontal biofilms [165]. Furthermore, when the light dose was increased to 54 and 108 J·cm^−2^, *A. actinomycetemcomitans* and *P. gingivalis* biofilms were completely eradicated, respectively [165]. Regarding periodontal regeneration, two in-situ curable biomaterials, BioM1 and BioM2, were developed as support for PS and had excellent mechanical and antimicrobial behaviors [15]. The BioM1 and BioM2 consisted of urethane dimethacrylate and a tri-armed oligoester-urethane methacrylate, respectively. The PS was composed of a mixture of β-tricalcium phosphate (β-TCP) microparticles and 20 wt % photosensitizer mTHPC (Figure 7B). The BioM2 + PS with a single laser-illumination (652 nm) caused total suppression of *P. gingivalis*, while BioM2 + PS with repeated irradiation reduced 3.1 log in CFU counts [15].

PS binding to various cationic peptide or polymers can enhance their loading into the bacterial cells and target periodontal pathogens (Figure 7C). It was confirmed that aPDT with chlorin e6 (Ce6) and BLC 1010 suppressed periodontopathogenic bacteria [166]. Ce6-(Lys)_5_-OH (Ce6-5K) conjugate broadened the spectrum of activity against periodontal pathogens, compared to the pure Ce6. Ce6-5K showed a strong killing effect on almost all oral bacteria tested, and showed at least six logs of killing for periodontal pathogens including *P. gingivalis*, *A. actinomycetemcomitans*, and *B. forsythus* [167]. In addition, two types of polylysin-porphycene conjugates GlamTMPn and BOHTMPn were synthesized to assess the development of resistance in bacterial cells [168]. The combination of polylysin-porphycene conjugates and bacterial cells was quite stable and showed an extremely high photosensitivity on *F. nucleatum* with 60 s irradiation [168]. Considering the precise targeting of aPDT, LiDps genetic construct was dual functionalized with SnCe6 and biotin (KLFC-LiDps-B-SnCe6) for *A. actinomycetemcomitans* biofilm-targeting, producing light-activated membrane disruption [169]. Streptavidin was used to couple biotinylated dodecamer to a biotinylated anti-*A. actinomycetemcomitans* antibody, which increased the loading efficacy of photosensitizer onto the bacterial cells. Light-induced activity of the targeted photosensitizer reduced the viability of *A. actinomycetemcomitans* biofilm [169]. In addition, a Fotolon sensitizer, composed of Ce6 and polyvinylpyrrolidone (PVP), obtained over 99.9% reduction in CFU in twenty Gram-positive and thirty Gram-negative clinical anaerobic strains. The PVP increased the quantum yield and fluorescence lifetime of Ce6, which contributed to the dispersion of particles and increased its bioavailability [170].

In recent years, nanoparticle-based PDT has become a hot research field in both tumor treatment and antibacterial therapy [171,172,173]. Extensive efforts had been made on how to disturb the formation of biofilms, and enhance the delivery of PS and its combination with bacteria to improve bactericidal efficiency by using the nanoparticles in oral diseases [51,174,175]. 

**Figure 8:**
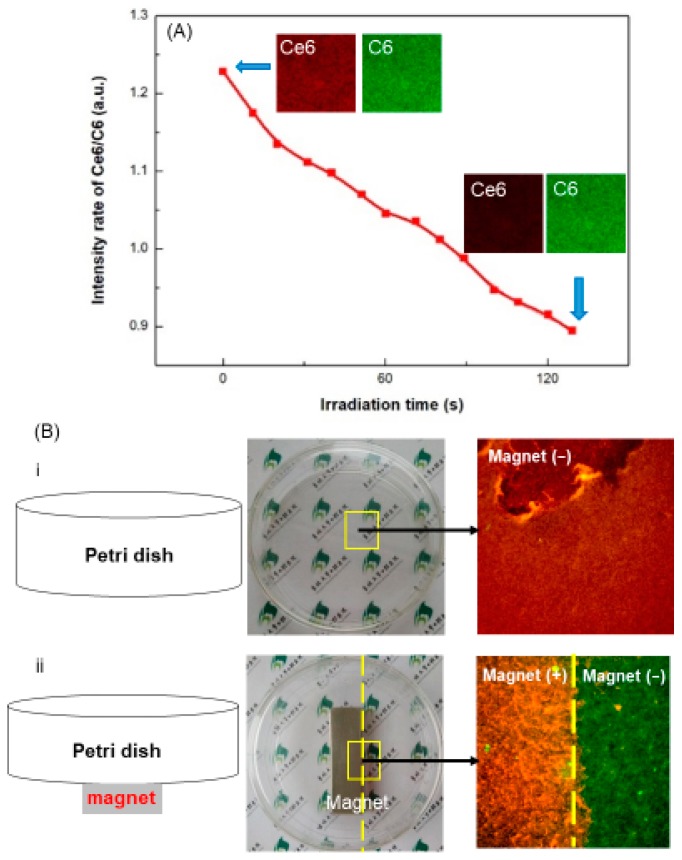
Real-time monitoring and magnetic targeting functions of multifunctional nanoparticles: (**A**) Ratio metric intensity of Ce6/C6 based on the grayscale value of the confocal images from different detection channels in the irradiation time 0–130 s. Inset: The first two and last two images for illustration of the Ce6 consumption. (**B**) Comparison of aPDT effect in the presence of Fe_3_O_4_-silane@Ce6/C6 magnetic nanoparticles (MNPs, 2.5 μM Ce6) with and without external magnetic field. (i) aPDT effect of Fe_3_O_4_-silane@Ce6/C6 MNPs without magnetic targeting: (Left) schematic diagram; (middle) photograph showed culture dish without the magnet; and (right) live/dead image in yellow pane. (ii) aPDT effect of Fe_3_O_4_-silane@Ce6/C6 MNPs with magnetic targeting: (Left) schematic diagram; (middle) photograph showed culture dish with the magnet; and (right) live/dead image in the yellow pane. Live bacteria were stained green. Dead bacteria were stained red. Samples without magnet showed primarily dead bacteria. Samples with magnet had primarily dead bacteria in the magnetic region. (Reproduced with permission from [177]. Elsevier, 2019.)

For intra-pocket administration, a major disadvantage of traditional nanoparticles carrying PS agents is the low delivery efficiency due to the drainage of gingival crevicular fluid and high saliva fluid turnover. Therefore, efficient delivery and penetration of the antibacterial agent to the exact site of infection is still highly desirable. In addition, real-time monitoring of the drug consumption and distribution is another issue that should be taken into consideration. The unknown effective dose of therapeutic agents reaching the disease site is an important uncertainty, and often leads to over-dosing or insufficient dosing [176]. In a recent study, a novel type of multifunctional nanoparticles Fe_3_O_4_-silane@Ce6/C6 was developed to trigger the aPDT for periodontal therapy, as shown in Figure 7D [177]. Fe_3_O_4_ and PS molecules were encapsulated with amphiphilic silane, which formed the hydrophobic interspace between the alkyl chains of silane and the octadecyl groups of oleic acid being bonded on the surface of Fe_3_O_4_ NPs. The PS molecules were well dispersed inside the nanocomposites. The hydroxyl of silane stretched out to enhance the water solubility. In addition, to solve the real-time monitoring issue, dye Coumarin 6 (C6) hydrophobic organic molecules were also encapsulated together inside the hydrophobic environment of silane via the hydrophobic interaction, without increasing the particle size. Besides the imaging function with the efficient green emission of C6, another advantage in this design was the real-time aPDT monitoring function [177]. The C6 molecule was not sensitive to red light (630 nm wavelength) but could be co-excited with Ce6 by 405 nm wavelength. Therefore, by visual imaging monitoring of the ratio of the fluorescence of Ce6/C6, the aPDT effect (Ce6 consumption) and distribution profile in periodontal pockets could be monitored in real time. This facilitated the optimization of therapeutic doses and treatment time-windows. Figure 8 shows the real-time monitoring and magnetic targeting functions of the multifunctional nanoparticles. The in vitro antibacterial experiments showed that three single-species periodontitis-related biofilm survival was reduced by about 4–5 orders of magnitude after 3 min of irradiation by the 630 nm light (100 mW·cm^−2^) [177]. Therefore, the multifunctional design has great potential for antibacterial applications to inhibit the occurrence and progression of periodontitis.

Visible light is usually used as the main light source to trigger the PS for periodontal therapy. Red light is used as the excited light source since it can penetrate deeper than violet, blue, green, or yellow lasers [178]. However, the limited penetration depth of visible light cannot reach deep periodontal pocket to disturb or clear the periodontal pathogens. There is a biological tissue window ranging from 700 nm to 1100 nm in the near-infrared (NIR) light. The excitation of NIR light provides a deeper penetration and lower autofluorescence, and reduces phototoxicity and photodamage side effects [179]. Titanium dioxide (TiO_2_) is widely used as a catalyst because of its high stability and good photocatalytic activity. ROS produced by photocatalysis of TiO_2_ has high bactericidal and sterilizing effects on oral pathogens [180]. However, one major concern about TiO_2_ application is that it can only be activated by ultraviolet (UV) light due to its wide band gap energy of 3.2 eV. Furthermore, the irradiation of UV light may cause DNA damage and cell death. Recently, various upconversion nanoparticles (UCNs) have been developed to solve this problem and are applied widely in cancer therapy. However, little research has been performed on antimicrobial and bacteriostasis. Under near-infrared light irradiation, the UCN core converts the low energy NIR light to high energy UV light, which induces the photocatalysis activity of TiO_2_ via an anti-Stokes emission process. Based on this mechanism, a recent study synthesized a core–shell structured NaYF_4_:Yb^3+^, Tm^3+^@TiO_2_ (TiO_2_-UCNs) which was applied in aPDT for periodontal pathogens (Figure 7E). The UCN cores, NaYF_4_:Yb^3+^, Tm^3+^, were synthesized by thermal decomposition in an oleic acid system [181]. Polyvinylpyrolidone (PVP) was added for the modification of the surface of UCNs. Subsequently, titanium n-butoxide served as a titanium precursor to coat the TiO_2_ shell on the surface of the UCNs under hydrothermal reactions. The results showed that TiO_2_-UCNs had excellent antibacterial activity against periodontal pathogens including *P. gingivalis* and *F. nucleatum*. Reductions of 4–5 orders of magnitude in bacteria CFU were achieved in the light groups. At 4 h after the initial irradiation of 2.5 W·cm^−2^ for 5 min, the suspension of bacteria was almost cleared when the concentration of drugs was at 2 mM. Therefore, NIR-triggered aPDT is a promising approach for effective periodontitis treatment. 

Despite of many publications on the use of aPDT to combat periodontal pathogens and biofilms, several problems remain. For instance, PS parameters, the strategy of surface modification and doses of PS, and irradiation intensity which determines the type and kinetics of bio-distribution and toxicity at the cellular and tissue level, should be strictly tested to ensure minimal toxicity. In addition, the efficient PS delivery, organ distribution, accumulation, retention, degradation, metabolism and clearance properties should be investigated at the organ level. It is essential for PS to enhance the selectivity of the pathogens without killing the benign microflora in oral environment. Another concern is that the irradiation intensity should be strictly and repeatedly tested before clinical application to avoid oxidative damage to the host cells. In addition, in vivo studies should be performed in animal models to confirm the feasibility for clinical applications. Further studies are needed to solve these problems for clinical application to be successful.

## 8. Conclusions

This article reviews cutting-edge research on antibacterial properties of novel polymeric materials against periodontal pathogens and oral biofilms. The focus was particularly on antibiotics delivery, functional GTR membrane, dental composite and bonding agents for decay restoration, and PS modification for aPDT enhancement. Due to increasing resistance development, there is a need to find delivery systems compatible with a diversity of antibacterial agents. Novel polymers offer meritorious opportunities and are compatible with a wide range of antibiotics to combat periodontitis. Endowing GTR membranes with antibacterial functions could not only benefit tissue regeneration, but also lower the risk of infection. In addition, the new generation of resins for Class-V restorations offers strong protein-repellent and antibacterial capabilities against periodontal biofilms, and is promising to improve a wide range of dental treatments. Furthermore, polymer modification of PS could increase the dispersion of PS and improve its bioavailability, thus enhancing the aPDT effects against periodontal pathogens. Therefore, the novel bioactive and therapeutic polymers have great potential for treating periodontal disease and other dental diseases.

## Figures and Tables

**Table 1:** Polymeric materials as drug delivery systems for combating periodontal pathogens.

Type	Polymer/Polymer-Based Product	Drug/Antibiotics	Periodontal Pathogens	References
Film	Chitosan	Chlirhexidine (Chx) gluconate Taurine (Amino acid)	*P. gingivalis*	[9]
	Cellulose acetate phthalate and Pluronic F-127 (CCAP)	Metronidazole	*>P. gingivalis*	[55]
	PLGA	Secnidazole (SC) Doxycycline hydrochloride (DH)	*P. gingivalis* *F. nucleatum*	[56]
Gel	Arestin^®^	Minocycline	*>A. actinomycetemcomitans*	[57]
	Polyester	Doxycycline hyclate Metronidazole	*>P. gingivalis*	[46]
	Badam gum Karaya gum Chitosan	Moxifloxacin	*>A. actinomycetemcomitans*	[58]
	Atridox^®^	Doxycycline hyclate	*P. gingivalis* *F. nucleatum*	[59,60]
Chip	PLGA	Chlorhexidine (CHX) CHX digluconate	*P. gingivalis*	[61]
Strip	Hydroxypropylcellulose	Green tea catechin	*P. gingivalis* Prevotella spp.	[62]
Cube	poly(glycerol sebacate) (PGS)	Berbereine chlorhexidine	*P. gingivalis* *A. actinomycetemcomitans*	[63]
Microparticles	Gelatin	Doxycycline	*P. gingivalis*	[64]
Nanoparticles	PLGA Polymersomes	Minicycline Metronidazole Doxicycline	*P. gingivalis* *T. forsythia* *T. denticola* *P. gingivalis*	[65,66]
	PEGylated PLGA	Minocycline	*A. actinomycetemcomitans*	[67]
	PLGA	H.madagascariensis leaf extract (HLE)	Prevotella species	[68]
